# Indents

**Published:** 1921-04

**Authors:** 


### INDENTS

**☞Brief Articles of Technical or Scientific Value will receive notice and comment. Contributions invited.**

---

Post-operative Pain. With regard to post-operative pain, it is significant that despite miscellaneous methods of operation, adverse conditions, and generally an unaseptic environment, the great majority of extractions are successful. However, since surgical exodontia is on the increase, pain is frequently a sequel. Certainly its alleviation should be a matter of vital concern to every one imbued with the proper spirit of his calling. Speaking of a successful operation in the face of unendurable pain is a felicitation shared by the surgeon alone; the patient is likely to give a "painful version" of that success. Having eliminated probable exaggeration, which is characteristic of certain individuals, we are confronted by bare facts which demand our serious attention.

Careful check-up of the anesthesia and operative technique will solve many problems. In my practice I find, as each and every step is studied and perfected, a proportionate percentage of operative sequelae is reduced. At present we estimate this percentage at three per cent., of which two per cent. complain of pain. Considering that we deal almost exclusively with surgical exodontia and general oral surgery, this percentage seems not out of proportion.

When searching for the cause of pain, we must be sure that we do not leave the most obvious for the last. The writer listened to a discussion upon hemicrania by several medical specialists, during which discussion the question of potentialities of teeth seemed to be a last consideration.

Post-operative pain may often be traced to simple mechanical trauma. Pain confined to the operative zone will frequently be traced to dressings that, although well designed, when left too long become a source of irritation, chiefly because they block drainage and interfere with union. Where co-existing conditions are ruled out the treatment is obvious.

Another simple condition, that we meet commonly as an aftermath of an otherwise satisfactory extraction, is the case of a cuspid or a molar area which apparently runs a normal course the first several days and on the fourth or fifth day the patient returns complaining of pain. Examination reveals a taut mucosa folded over an angular or irregular hard substratum, blanched in part and having an angry red appearance at the periphery. The slightest palpation elicits severe pain. Relief in this case may be obtained by retracting the flaps (under conduction anesthesia), trimming (with Ronguer forceps) the projecting or ragged alveolus and dressing lightly. Where the pain is due to the involution of the gum tissue over alveolar borders that are otherwise satisfactory, separating the bucco-lingual flaps by means of a superficial dressing so that contact with the alveolar edge is obviated will tide the patient over.

Still another condition, even more trying, always accompanied by a history of local and referred pain, loss of sleep and general irritability, is traced to a traumatized tooth adjacent to the wound. The operative history will probably show the vigorous use of an elevator or dislocation accidental to extraction. Slightest percussion of the offending tooth will give both subjective and objective proof of its involvement. Electro-dental testing will help to determine the vitality and normality of the pulp. This may be verified by the thermic and ethyl chlorid tests. Radical treatment will be indicated if the pulp is found devitalized. Temporarily this condition can be relieved by opening into the tooth and removing the dead pulp. The reduction of the occlusion in any event and ligation of the tooth wherever possible, plus the use of the ice pack, will afford relief.

A not infrequent condition met with by the general practitioner is the protracted pain in edentulous jaws which is usually associated with remote neuralgias. Examination reveals a history of extraction probably months before, with pain, sleeplessness, and not infrequently emaciation dating back to the time of the operation. The clinical picture presents a particularly healed, irregularly shaped alveolar crest containing numerous bumps and grooves, some of the latter probably hemorrhagic. Occluding the jaws will show that the opposing teeth are the offenders. In connection with these cases, it is a curious fact that the dentist commonly advises waiting for the "gums to heal" before he will be ready to insert artificial teeth. To the contrary, in cases where owing to extractions the gingiva has been rendered vulnerable to attack by any agency, especially the opposing teeth, immediate "shoeing" by means of base plates is recommended. Patients who can afford the added expense are advised to have a "temporary" set without delay. In the light of recent developments in plate technique, the dentist should have no difficulty in retaining the denture. We find that occasional compensating for the atrophy of tissue by means of Kerr's compound is a simple procedure and the patient may be thus afforded great comfort; incidentally a more even healing is assured.

A most disquieting condition to both surgeon and patient is the protracted anesthesia mesial to the site of the operation—an evidence of sensory nerve injury. The patient may describe this condition as a burning, pinching, tingling and generally uncomfortable sensation. This is commonly seen in the mandible, although similar trouble has occurred in the zone covered by the infra-orbital injection. Stimulation of the part is essential. The violet ray, hot irrigation and massage are found helpful. In rare cases re-operation may be the only remedy.

Infected wounds will manifest that condition by the presence of pus or putrescent odor, or sloughed tissue, tenderness to palpation, glandular involvement, coated tongue, foul breath, headache, etc. Generally this condition is subacute. In the acute state, local pain may be severe; septic temperature and a leukocytosis is to be looked for. In the latter case free drainage, stimulating irrigations, the application of cie packs one-half hour alternately, hot foot-baths, purgatives, liquid diet and rest is to be advised. In the quiescent state, removal of the necrotic tissue, proper drainage and frequent warm stimulating irrigations are indicated.

Trismus, in addition to severe pain, is not uncommon as a sequel to extensive surgery attending the removal of first and third impacted molars, yet a reaction not abnormal to operations of this nature. Physiological rest is an outstanding requirement in the treatment of these cases. The use of ice packs, liquid diet and the internal administration of acetanilidi, grs. ij; phenacetini, grs. v; caffeinae citrat, gr. j, to be made up in powders or capsules and taken four or five hours apart until pain is relieved, have been found effective.

In every case where the operative data is not clear, it is advisable to radiograph the part, and if possible, in addition to an extra-oral plate, an intra-oral film should be taken. In refractive cases the presence of a retained root or granuloma should be looked for. Where the findings are positive, re-operation at the earliest moment is advisable. Until then, treatment as outlined for the expectant plan is to be followed.

Since our understanding of infection and immunity is becoming clearer, we are able to account for the marked systemic reaction that often follows the surgical interference or removal of an infected tooth; a condition significant of the lighting up of closed focal infection. This may occur not only in operations upon diseased teeth, but also in cases of extraction of numerous healthy teeth, but of a pyorrhetic environment. The absorption of toxins into the general system leads to toxemia. Where secondary foci are present these may become activated and a general violent reaction, characterized by all symptoms of an acute septic condition, may ensue. To the pathologist such a reaction is of value as a therapeutic test. Generally, there is a variable temperature, pain markedly all over the body, a pounding at the back of the head, lassitude, malaise, loss of appetite, etc., lasting from three to six days, after which the patient recovers rapidly.

In point of caution, it should be said that the lighting up of too many foci may also lead to the absorption of bacteria and consequent bacteriemia. Since dental foci of infection usually harbor streptococci, the danger of streptococcemia, a very grave condition, should always be a deterring factor in undertaking too extensive septic mouth operations.—Extracts from *Dental Cosmos.*

---

DON'T USE AMMONIA TO REMOVE SILVER NITRATE STAINS. We find the following recommendation in one of the recent dental magazines. Do your investigations confirm the correctness of this method?

"Removal of Silver Nitrate Stain from Tooth—Use nitrate of silver, but as that stains the teeth I change the nitrate into iodid of silver by the action of iodin; this in turn may be discharged by ammonia, leaving the tooth stainless."

*Answer:* This department investigated the subject of your query sometime ago. The method you mention was tried. It is absolutely useless, unless the stain is merely superficial.

The trouble with silver nitrate stains is that if the dentist believes he has a method of removing the stain later, he is apt to apply the silver nitrate rather freely. The staiaing depends upon the strength of the solution, the quantity used, and the length of time it is in contact with the tooth. If the dentist applies too much, or too strong a solution, or leaves it on the tooth too long, then the stain will penetrate deep into the tooth, and there will be no feasible way either to control extent of penetration or to remove deep .stains. The best method is to *prevent* objectionable staining by application of our cavity lining to all parts of the tooth that should be protected. After this protective coating has served its purpose, it can be removed at once with cavity lining solvent.—Department of Chemistry, *Caulk's Dental Quarterly.*

---

How to Make Your Own Flux. Fill a bottle with water.

Then pour the water into a glass and put the glass in a pan. Look in a text-book and find out how much borax and boracic acid will make a saturate solution in the quantity of water you intend to use. Then excuse yourself to the lady in the waiting-room while you go down to the drug store to buy the borax and boracic acid. When you get back, look and see if your patient is still there. If she is, carefully throw the water down the sink, put the pan back in the kitchen, place the chemicals where you can't find them again, phone your depot for a bottle of Caulk's soldering fluid and a can of Caulk's soldering wax (which has flux in it), put on your white coat, wash your [illegible]nds, and do some of the work that no one else can do for you.

